# Supplementary material for: Gut microbiota‐CRAMP axis shapes intestinal barrier function and immune responses in dietary gluten‐induced enteropathy
Source: EMBO Mol Med. 2021 Jun 14;13(8):e14059. doi: 10.15252/emmm.202114059 (PMC8350901; doi:10.15252/emmm.202114059)
Supplement: Supplementary file 1 — Appendix [file EMMM-13-e14059-s009.pdf]

Gut microbiota-CRAMP axis shapes intestinal barrier function and immune responses in dietary gluten-induced enteropathy

Appendix

Table of contents

Contents	Pages
Appendix Table S1: The characteristics of human subjects	2
Appendix Table S2: Primers used for RT-qPCR	3
Appendix Supplementary Methods	4-9

Appendix Tables

Appendix Table S1: The characteristics of human subjects (no. or mean ± SD). Related to Materials and Methods.

**Variables**	**Double Negative (*n*=50)**	**Anti-tTG IgA^+ (*n*=46)**	**Anti-DGP IgG^+ (*n*=50)**	**Double Positive (*n*=13)**
Age, yr	18.72±1.11	18.61±0.94	19.06±1.34	18.7±0.57
Men, n (%)	45 (90.0)	29 (63.0)	25 (50.0)	3 (23.1)

Anti-Ttg IgA: anti–tissue transglutaminase immunoglobulin A antibodies

Anti-DGP IgG: anti-deamidated gliadin peptides immunoglobulin G antibodies

Appendix Table S2: Primers used for RT-qPCR. Related to Materials and Methods.

Gene	Forward	Reverse
Il15	5'-ACATCCATCTCGTGCTACTTGTA-3'	5'-GCCTCTGTTTTAGGGAGACCT-3'
IL15	5'-TTGGGAACCATAGATTTGTGCAG-3'	5'-GGGTGAACATCACTTTCCGTAT-3'
Ifng	5'-ATGAACGCTACACACTGCATC-3'	5'-CCATCCTTTTGCCAGTTCCT-3'
Il6	5'-TAGTCCTTCCTACCCCAATTTCC-3'	5'-TTGGTCCTTAGCCACTCCTTC-3'
Il8	5'-GACTCTTGCGTCAACTTCAAGG-3'	5'-CAGGCTGTCTTTTGTCAACGA-3'
Il27	5'-CTGTTGCTGCTACCCTTGCTT-3'	5'-CACTCCTGGCAATCGAGATTC-3'
Ccl2	5'-TTAAAAACCTGGATCGGAACCAA-3'	5'-GCATTAGCTTCAGATTTACGGGT-3'
COX2	5'-GGGTGTGAAGGGAAATAAGG-3'	5'-TGTGATTTAAGTCCACTCCATG-3'
Ifna	5'-GCAATCCTCCTAGACTCACTTCTGCA-3'	5'-TATAGTTCCTCACAGCCAGCAG-3'
Ccl5	5'-GCTGCTTTGCCTACCTCTCC-3'	5'-TCGAGTGACAAACACGACTGC-3'
Il23	5'-ATGCTGGATTGCAGAGCAGTA-3'	5'-ACGGGGCACATTATTTTTAGTCT-3'
Il2	5'-TGGTTTGCCATCGTTTTGCTG-3'	5'-ACAGGTGAGGTTCACTGTTTCT-3'
P.aeruginosa	5'-CTGGGTCGAAAGGTGGTTGTTATC-3'	5'-GCGGCTGGTGCGGCTGAGTC-3'
A. muciniphila	5'-CAGCACGTGAAGGTGGGGAC-3'	5'-CCTTGCGGTTGGCTTCAGAT-3'
lasB	5'-GTCGACTCTAGAGGATCCCCTGGCCCCTCGCTGAGCGC-3'	5'-AGAATTCGAGCTCGGTACCCCTGGCGGAAGACGGCTTGAGC-3'
16S	5'-GCAGGCCTAACACATGCAAGTC-3'	5'-CTGCTGCCTCCCGTAGGAGT-3'
Actb	5'-CCCAGGCATTGCTGACAGG-3'	5'-TGGAAGGTGGACAGTGAGGC-3'

Appendix Supplementary Methods

The isolation of IECs, IELs and LP

IECs, IELs an LP cells were extracted from the small intestine. Briefly, the small intestine was collected and the Peyer patches were removed. Intestines were opened longitudinally, cut into pieces of 5 mm, and washed extensively in ice-cold Hank’s Balanced Salt Solution wash solution (HBSS; $Mg2^{+}$- and $Ca2^{+}$-free; Beyotime, Shanghai, China). IECs and IELs cells suspension were then collected by twice incubations of intestinal pieces for 20 minutes at 37°C in HBSS containing 5 mM Ethylenediaminetetraacetic acid, 1 mM Dithiothreitol and 10% fetal calf serum. IECs was separated from IELs in supernatant through Percoll (Sigma, Shanghai, China). Intestinal pieces were then washed in RPMI 1640 medium containing 10% fetal calf serum, then cut into small pieces and incubated for 30 minutes at 37°C with a solution of RPMI 1640 medium containing 1 $mg{\cdot}mL^{-1}$ DNase (Sigma, Shanghai, China) and 1 $mg{\cdot}mL^{-1}$ collagenase type IV (Sangon Biotech, Shanghai, China) to obtain a homogeneous LP cells suspension.

Microbiota composition analyses

Total DNA was extracted from duodenal content using the Fast DNA Spit Kit for Feces (MP Biomedicals, Shanghai, China) according to the manufacturer’s instructions. The PCR amplification products were separated by 1.0% agarose Gel electrophoresis, and used the Gel/PCR Extraction Kit (BIOMIGA, CA, USA) to recover the target fragments. DNA was extracted from samples and amplified for the hypervariable 16S rRNA gene v3 to v4. Products were then sequenced using the Illumina MiSeq platform (Jiangsu,

China).

Antibodies for western blot

Antibody for CRAMP pAb (1:1,000, PA-CRPL-100) was purchased from Innovagen AB (Lund, Sweden). claudin-1 pAb (1:5,000, ab180158), occludin pAb (1:1,000, ab216327), zonulin pAb (1:10,000, ab131236) TRAF6 pAb (1:5,000, ab40675) and p65 pAb (1:50,000, ab32536) were purchases from Abcam (Cambridge, UK). Antibodies for p-AKT mAb (4060S), Akt mAb (4685S), MyD88 mAb (4283S) and p-p65 mAb (3033S) (all in 1:1,000) were purchased from Cell Signaling Technology (MA, USA). Antibodies for ZO-1 mAb (1:500, 40-2200) and ZO-2 pAb (1:250, 71-1400) were purchased from ThermoFisher Scientific (MA, USA). Antibody for β-actin mAb (1:200,000, AC026) was purchased from Abclonal Technology (Hubei, China).

Preparation of pepsin/trypsin digested (PT)-gliadin

PT-gliadin was prepared by enzymatic digestion as described previously (Frazer *et al*, 1959). Briefly, 5 g of gliadin (Sigma, Shanghai, China) was dissolved in 50 mL of 0.2 N HCl for 2 h at 37°C with 0.1 g of pepsin (Sigma, Shanghai, China). The resultant peptic digest was further digested by the addition of 0.1 g of trypsin (Sigma, Shanghai, China) after the pH was adjusted to 7.4 using 2 M NaOH. The solution was stirred vigorously at 37°C for 4 h, boiled (100°C) for 30 min, freeze-dried, lyophilized in 10-mg aliquots, and stored at -20°C until use.

Gut permeability assay

For assessment for gut permeability, 4 hours fasted mice received 500 $mg\cdot kg^{-1}$ FITC-Dextran 4000 (Sigma, Shanghai, China) intragastrically and blood collected 4 hour later.

Serum concentration of FITC-dextran was determined by fluorometry (excitation 485 nm; emission 520 nm on a FLUOstar Optima microplate reader; BMG Labtech) relative to standard curve generated from serial dilution of FITC-dextran in control (non-treated) serum.

Flow cytometry

Single cell suspensions were stained for 30min at 4 ℃ after FcγRII/III blocking (Miltenyi Biotec, Bergisch Gladbach, Germany). Cell suspensions were then incubated for 15 min at room temperature in PBS with following antibodies: Alexa Fluor® 700 anti-mouse CD45 (103128), Brilliant Violet® 711 anti-mouse CD11c (117349), Brilliant Violet® 605 anti-mouse CD11b (101257), APC anti-mouse CD103 (121413), Brilliant Violet® 421 anti-mouse CX3CR1 (149023), Brilliant Violet® 421 anti-mouse CD3e (100341), PE anti-mouse CD314 (NKG2D) (130207), APC anti-T-bet (644813), Alexa Fluor® 488 anti-mouse FOXP3 (126405) (BioLegend, CA, USA), PE anti-mouse ROR GAMMA (T) (B2D) (12-6981-80) and PE-Cy™7 anti-mouse CD4 (552775; BD biosciences, MD, USA). Gating methods of fluorescence-activated cell sorting were programmed as $CD3^+CD4^+NKG2D^+$ (for IELs), $CD45^+CD11b^+CX3CR1^+$ (for macrophages) and $CD45^+CD11c^+CD11b^{+/-}CD103^{+/-}$ (for dendritic cells), $CD3^+CD4^+Foxp3^+$ (for Tregs), $CD3^+CD4^+$T-bet$^+$ (for Th1) and $CD3^+CD4^+$ROR-γt$^+$ (for Th17). The presence of cells displaying aldehyde dehydrogenase activity (ALDH cells) was determined using an ALDEFLUOR staining kit (StemCell Technologies, Vancouver, Canada). Stained cells were analyzed on an Attune NxT flow cytometer (Thermo Fisher Scientific, MA, USA) and data were analyzed using Flow Jo (10.6.2)

software (BD Bioscience).

Enzyme-linked immunosorbent assay (ELISA) assays

The levels of CRAMP and LL-37 were analyzed using the mouse CRAMP /human LL-37 ELISA kit (My biosource, Inc., CA, USA).

Histology and immunostaining

Fresh small intestine samples were fixed in 4% paraformaldehyde overnight, washed with running water for 2 h, rehydrated with gradient ethanol, and then embedded in paraffin. The Skiving Machine Slicer (RM2245; Leica, Hessen, Germany) diced 5 μm sections were stained with hematoxylin and eosin (H&E) following the standard procedure. For immunofluorescent staining, the following primary antibodies were applied: anti-CRAMP pAb (1:50, 12009-1-AP; Proteintech, Hubei, China), anti-E-cadherin pAb (1:100, 610181; BD Biosciences, MD, USA), anti-F4/80 mAb and anti-Ly6G mAb (1:100, ab6640, ab25377; Abcam, Cambridge, UK). Anti-rabbit AlexaFluor® 555 (CRAMP), anti-mouse AlexaFluor® 488 (E-cadherin) and anti-rat AlexaFluor® 488 (F4/80 and Ly6G) polyclonal secondary antibodies (all in 1:500, Invitrogen, Shanghai, China) were applied. The DAPI (4′,6-diamidino-2-phenylindole) staining was conducted according to the protocol provided by the manufacturer (Beyotime, Shanghai, China). A digital slice scanner (3DHISTCH Ltd, Budapest, Hungary) was used for H&E morphology evaluation. An ultra-high-resolution confocal microscope (LSM880, Carl Zeiss, Baden-Württemberg, Germany) was used for acquisition of immunofluorescence staining. ZEN software (Carl Zeiss) was applied for image processing.

Immunoblotting

Small intestine samples were homogenized in ice-cold lysis buffer RIPA (containing protease inhibitors and phosphatase inhibitors). Protein concentrations were determined by using a BCA Protein Assay Kit (Beyotime, Shanghai, China). Equal amounts of protein were electrophoretically separated in SDS–polyacrylamide gels and then transferred onto PVDF membranes (Millipore, MA, USA). The membranes were blocked with 5% w/v nonfat dry milk in TBS-T for 1 h at room temperature, further incubated with appropriately diluted primary antibodies overnight at 4 °C and probed with secondary peroxidase-labeled antibody for 2 h at room temperature. The proteins were visualized by Plus-enhanced chemiluminescence using FluorChem FC3 (ProteinSimple, CA, USA). The densitometric analyses of protein expression by Western blot were performed by AlphaView Software (ProteinSimple).

SCFA measurement by Gas Chromatography–Mass Spectrometry

The short-chain fatty acids (SCFAs) - acetate, propionate and butyrate, present in the fecal were analyzed by Gas Chromatography–Mass Spectrometry (GC/MS). Feces samples (50 mg) were collected directly into sterile tubes from live mice and were first homogenized in 500 μL of saturated NaCl solution. Thereafter, fecal samples were acidified with 40 μL 10% sulfuric acid. 0.8 mL diethyl ether was added to the sample to extract SCFAs. Samples were then centrifuged at 14,000 rpm for 15 min at 4 °C and the supernatants were used for the analyses of the SCFAs. Supernatants were injected into Rtx-WAX capillary column (30 m×0.25 mm×0.25 μm; Shimadzu, Kyoto, Japan) installed on the GC and coupled to the MS detector of GCMS-QP2010 (Shimadzu).

The initial oven temperature was 100 °C and increased to 140 °C at a rate of 7.5 $°C \cdot min^{-1}$. The temperature was further increased to 200 °C at a rate of 60 $°C \cdot min^{-1}$ and remained for 3 min. Helium was utilized as the carrier gas at a flow rate of 0.89 $mL \cdot min^{-1}$, and the column head pressure was 62.7 kPa. The injector was set at 240 °C. The injection mode was split and the ratio was 10:1. For mass spectrometer, ion source temperature was 220 °C, interface temperature was 250 °C, and the scan range was from m/z 2 to 100. Real time analysis software GCMS Postrun (GCMS solution Version 2.72, Shimadzu) was employed to compare the relative concentrations of SCFAs.
