# Supplementary material for: Gut microbiota‐CRAMP axis shapes intestinal barrier function and immune responses in dietary gluten‐induced enteropathy
Source: EMBO Mol Med. 2021 Jun 14;13(8):e14059. doi: 10.15252/emmm.202114059 (PMC8350901; doi:10.15252/emmm.202114059)
Supplement: Supplementary file 2 — Expanded View Figures PDF [file EMMM-13-e14059-s005.pdf]

Expanded View Figures

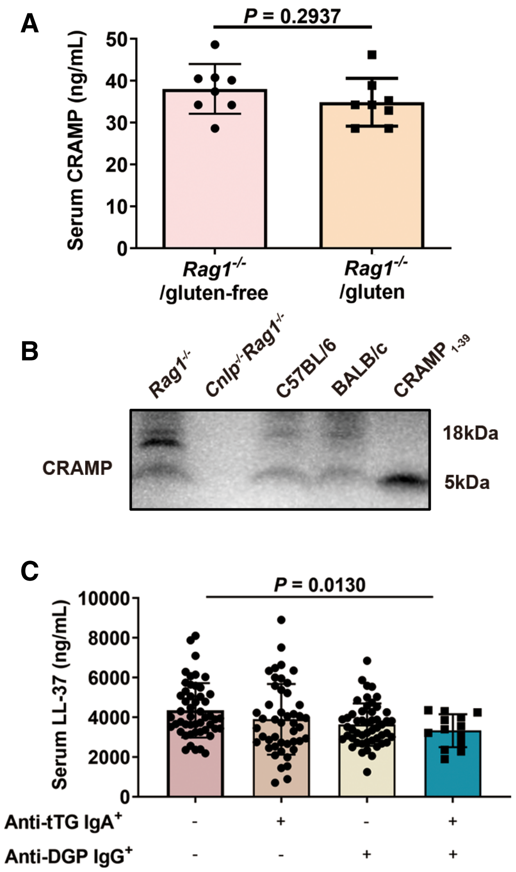

Figure EV1. The production of cathelicidin (CRAMP in mice and LL-37 in humans) in mice and in human subjects. Related to Fig 1.

- A Serum CRAMP determination by ELISA. *Rag1*$^{-/-}$/gluten-free: unsensitized *Rag1*$^{-/-}$mice fed with gluten-free diet. *Rag1*$^{-/-}$/gluten: unsensitized *Rag1*$^{-/-}$ mice fed with gluten-containing diet (n = 8).
- B Western blot of pro-form CRAMP and the mature peptide in duodenum of *Rag1*$^{-/-}$, *Cnlp*$^{-/-}$*Rag1*$^{-/-}$, C57BL/6 and BALB/c mice.
- C Serum LL-37 determination by ELISA. Anti-tTG IgA: anti-tissue transglutaminase immunoglobulin A antibodies. Anti-DGP IgG: anti-deamidated gliadin peptides immunoglobulin G antibodies (Anti-tTG IgA^{-}Anti-DGP IgG^{-}, n = 50; Anti-tTG IgA^{+}Anti-DGP IgG^{-}, n = 46; Anti-tTG IgA^{-}Anti-DGP IgG^{+}, n = 50; Anti-tTG IgA^{+}Anti-DGP IgG^{+}, n = 13).

Data information: Data (A, C) were representative and were the mean ± SD from three independent experiments. Data in B were representative from three independent experiments. *P* values were calculated by unpaired two-tailed *t*-test for comparison of two groups (A) or one-way ANOVA followed by Tukey's *post hoc* test for multiple comparisons (C).
Source data are available online for this figure.

Figure EV2. Replenishing CRAMP is protective against jejunal and ileal barrier damage during GIE. Related to Fig 3.

- A, B CRAMP determinations in (A) jejunum and (B) ileum by ELISA (n = 6).
- C, D Representative images of (C) jejunal and (D) ileal damage by H&E staining. Scale bar: 200 μm. The graphs depicted the ratio of the morphometric assessment of villus height to crypt depth (n = 6).
- E, F Western blot and densitometry analyses of tight junction proteins (claudin-1, occludin, ZO-1 and ZO-2) in (E) jejunum and (F) ileum (n = 4).

Data information: Data were representative and were the mean ± SD from three (A–D) or two (E, F) independent experiments. *P* values were calculated by one-way ANOVA followed by Tukey's *post hoc* test for multiple comparisons.
Source data are available online for this figure.

© 2021 The Authors

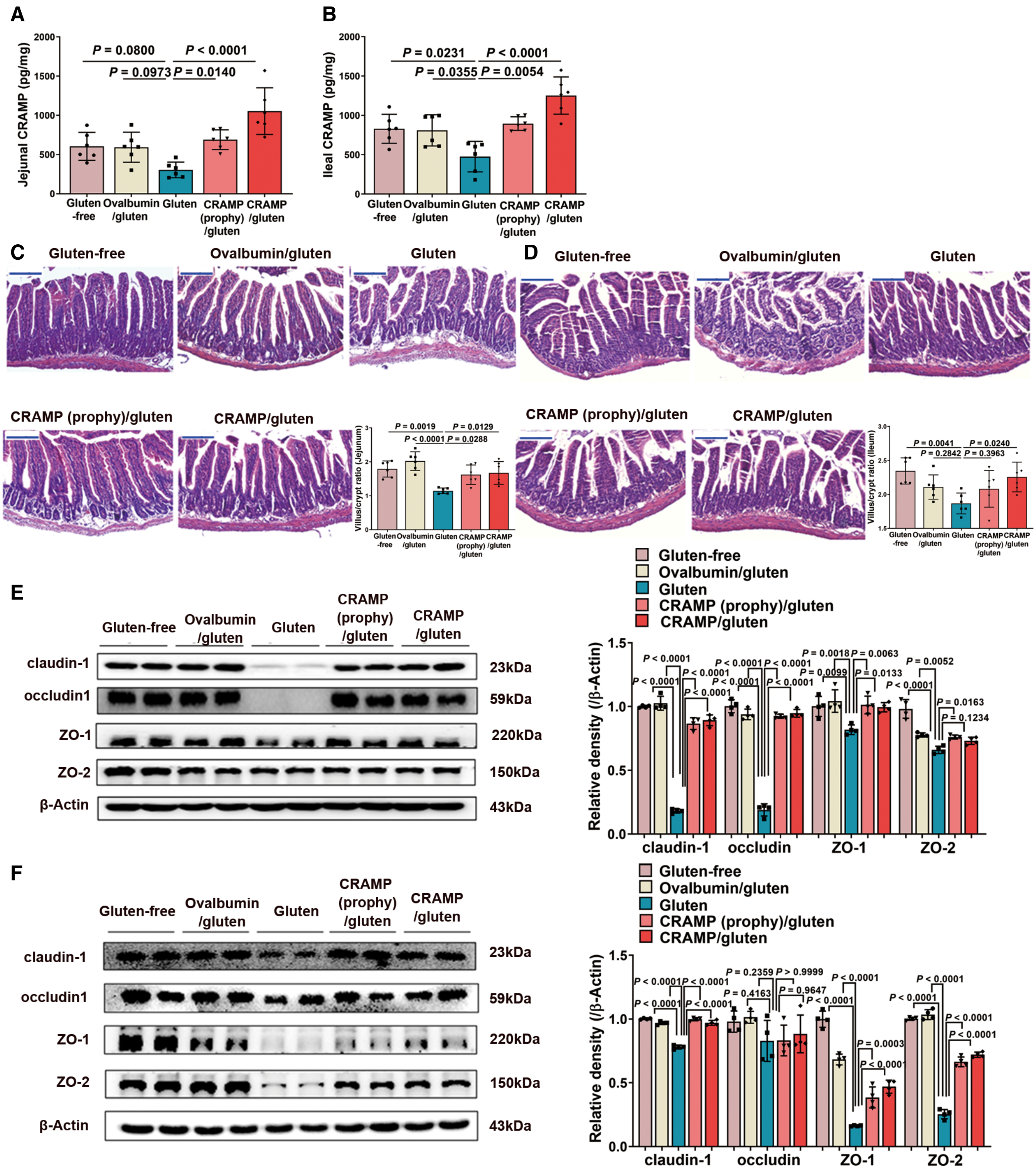
A
Jejunal CRAMP (pg/mg)
P = 0.0800
P < 0.0001
P = 0.0973
P = 0.0140
Gluten -free
Ovalbumin /gluten
Gluten
CRAMP (prophy) /gluten
CRAMP /gluten
B
Ileal CRAMP (pg/mg)
P = 0.0231
P < 0.0001
P = 0.0355
P = 0.0054
C
Gluten-free
Ovalbumin/gluten
Gluten
CRAMP (prophy)/gluten
CRAMP/gluten
Villus/crypt ratio (Jejunum)
P = 0.0019
P = 0.0129
P < 0.0001
P = 0.0288
D
Villus/crypt ratio (Ileum)
P = 0.0041
P = 0.0240
P = 0.2842
P = 0.3963
E
claudin-1
occludin1
ZO-1
ZO-2
β-Actin
23kDa
59kDa
220kDa
150kDa
43kDa
Relative density (/β-Actin)
claudin-1
occludin
ZO-1
ZO-2
F

Figure EV2.

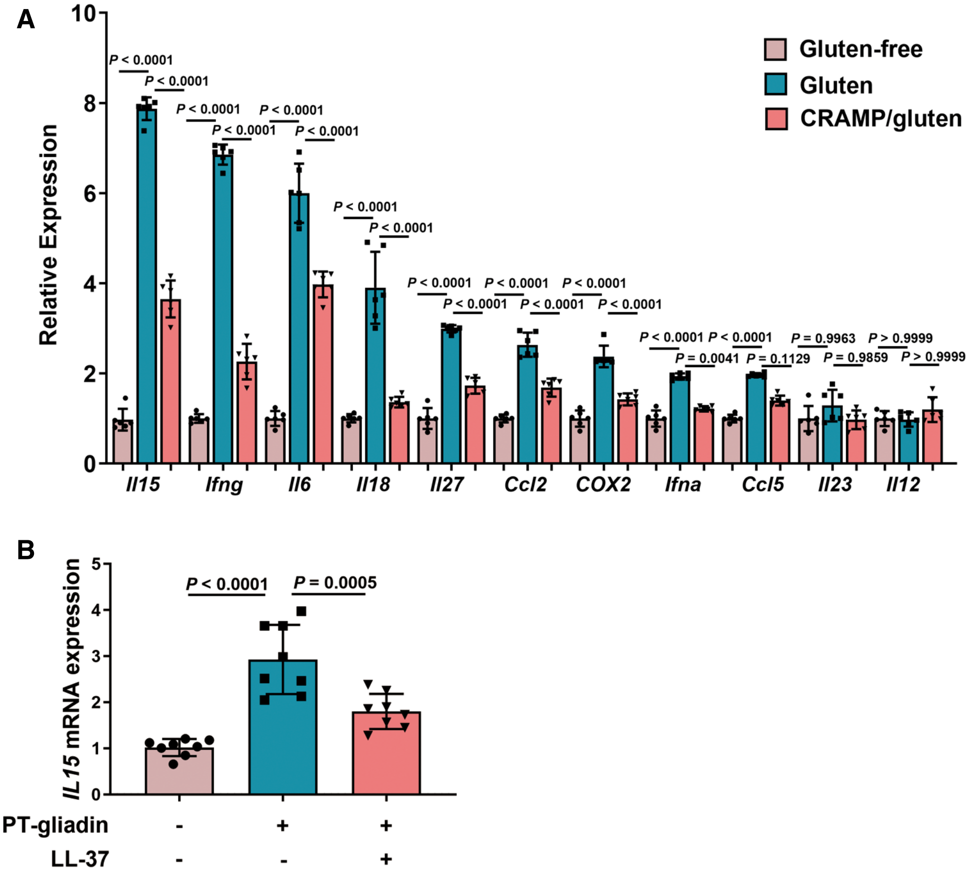

Figure EV3. Cathelicidin regulates cytokine expression in GIE and IL-15 expression in PT-gliadin-stimulated human epithelial cells. Related to Fig 5.

A The mRNA levels of cytokines in duodenum were measured by RT–qPCR ($n = 6$).

B The mRNA levels of *IL15 in vitro* were measured by RT–qPCR ($n = 8$).

Data information: Data were representative and were the mean ± SD from three (A) or four (B) independent experiments. *P* values were calculated by one-way ANOVA followed by Tukey's *post hoc* test for multiple comparisons.
Source data are available online for this figure.

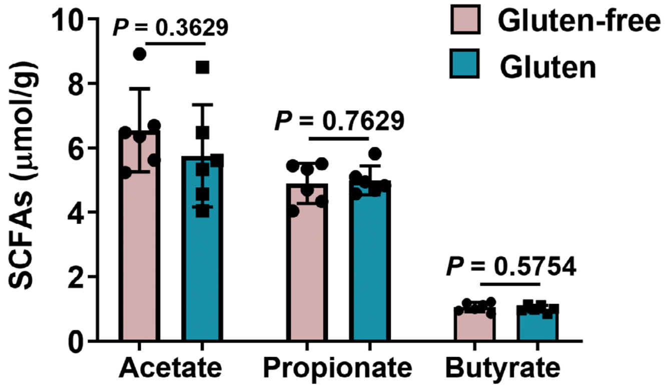

Figure EV4. Faecal content of short-chain fatty acids in GIE. Related to Fig 6.

Faecal levels of short-chain fatty acids (acetic acid, propionic acid and butyric acid) were measured by gas chromatography–mass spectrometer ($n = 6$). Data were representative and were the mean ± SD from three independent experiments. *P* values were calculated by unpaired two-tailed *t*-test for comparison of two groups.

Source data are available online for this figure.

© 2021 The Authors

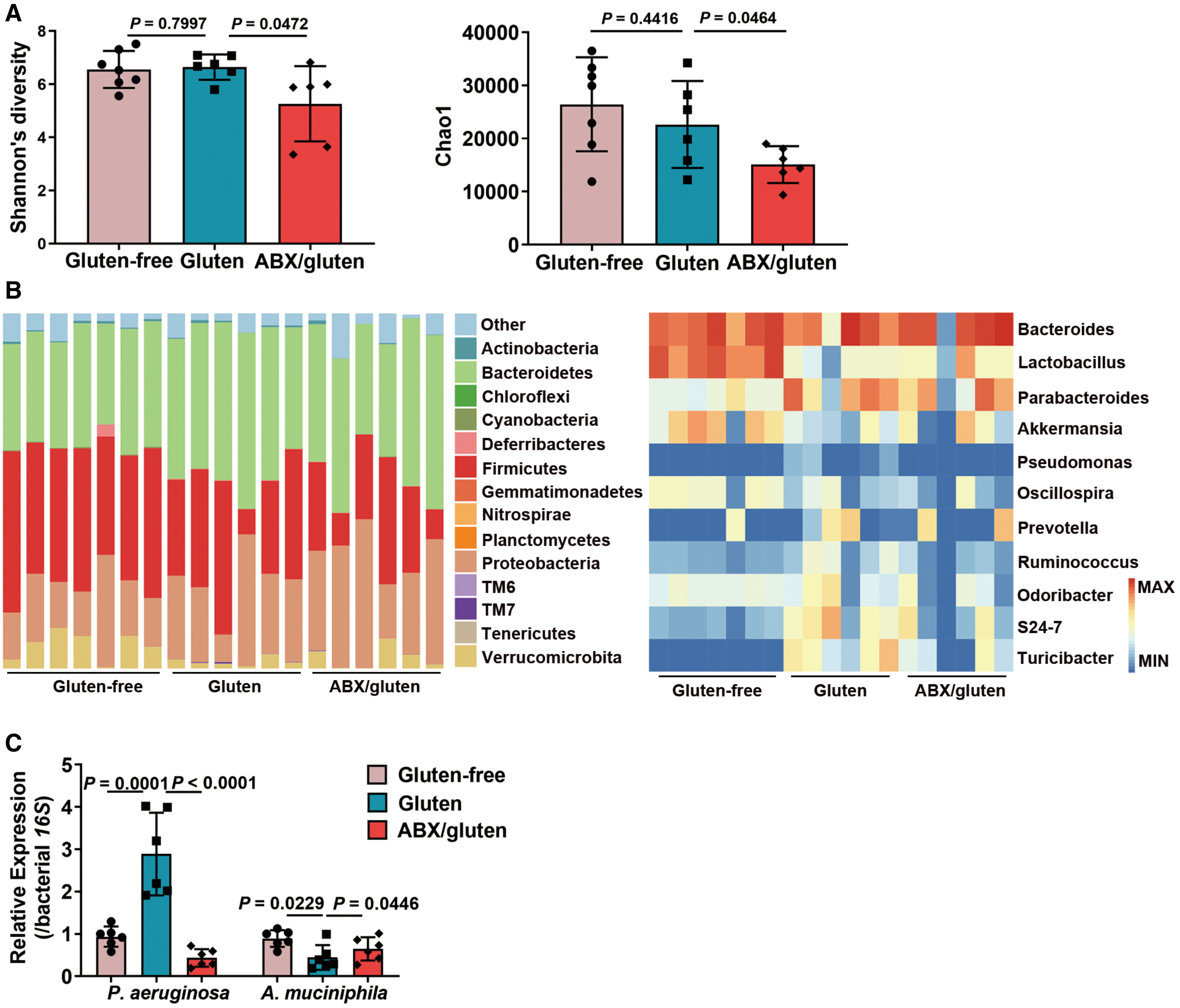

Figure EV5. The antibiotic cocktail treatment modulates gut microbiota composition, causing reduced *P. aeruginosa* and increased *A. muciniphila* during GIE. Related to Fig 7.

A Strain diversity (Left, Shannon's diversity; Right, Chao1 index; Gluten-free, $n = 7$; Gluten, $n = 6$; ABX/gluten, $n = 6$).

B The taxonomic composition distributions at phylum (left) and genus (right) levels (Gluten-free, $n = 7$; Gluten, $n = 6$; ABX/gluten, $n = 6$).

C Duodenal colonizing *P. aeruginosa* and *A. muciniphila* as determined by RT–qPCR using bacterial-specific species gene primers ($n = 6$).

Data information: Data in A were representative and were the mean ± SD. Data in C were representative and were the mean ± SD from three independent experiments. *P* values were calculated by one-way ANOVA followed by Tukey's *post hoc* test for multiple comparisons.
Source data are available online for this figure.
