# Supplementary figures and images for: Gut microbiota‐CRAMP axis shapes intestinal barrier function and immune responses in dietary gluten‐induced enteropathy
Source: EMBO Mol Med. 2021 Jun 14;13(8):e14059. doi: 10.15252/emmm.202114059 (PMC8350901; doi:10.15252/emmm.202114059)

Figure 1C

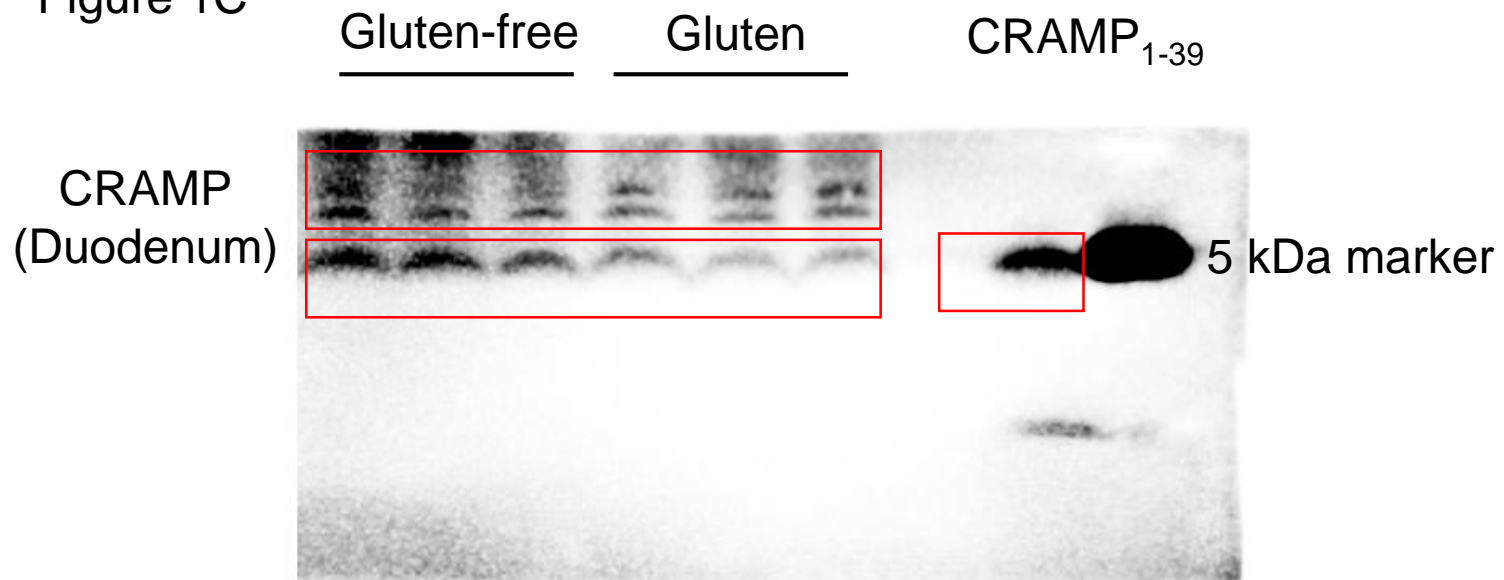

Figure 1D

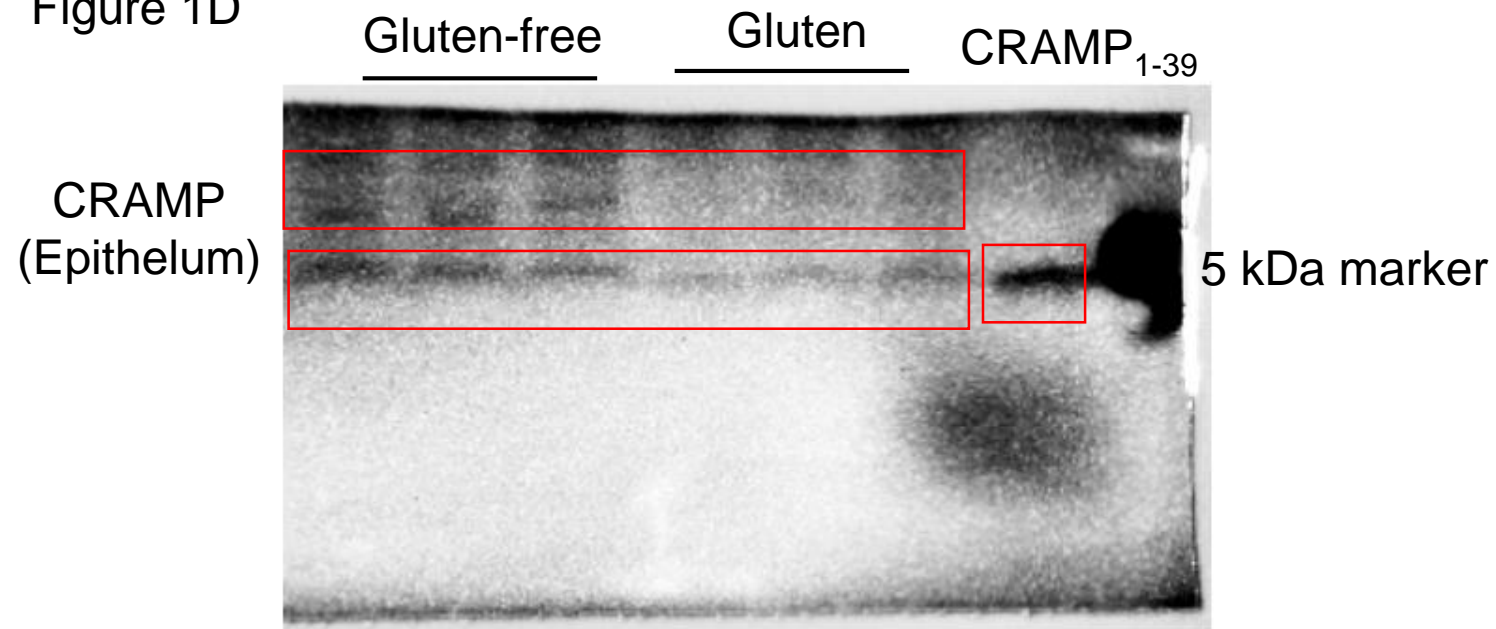

Figure 1E  
Gluten-free

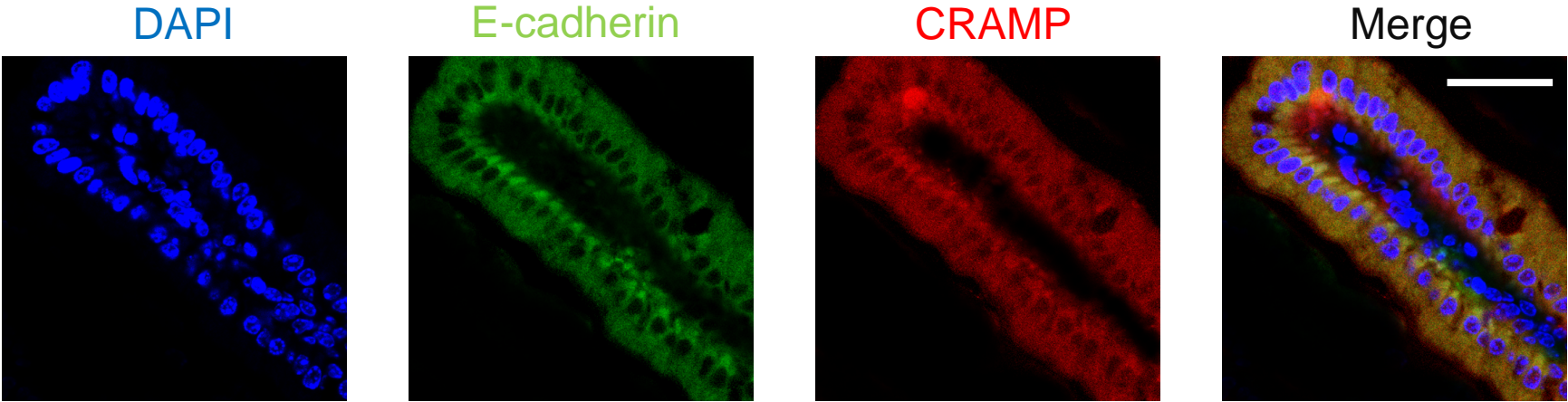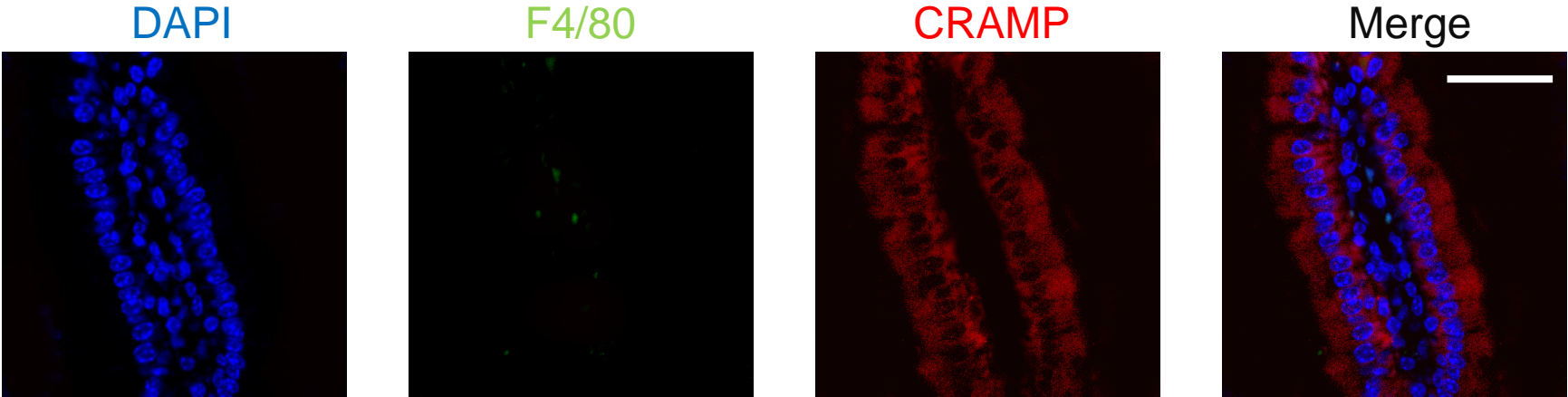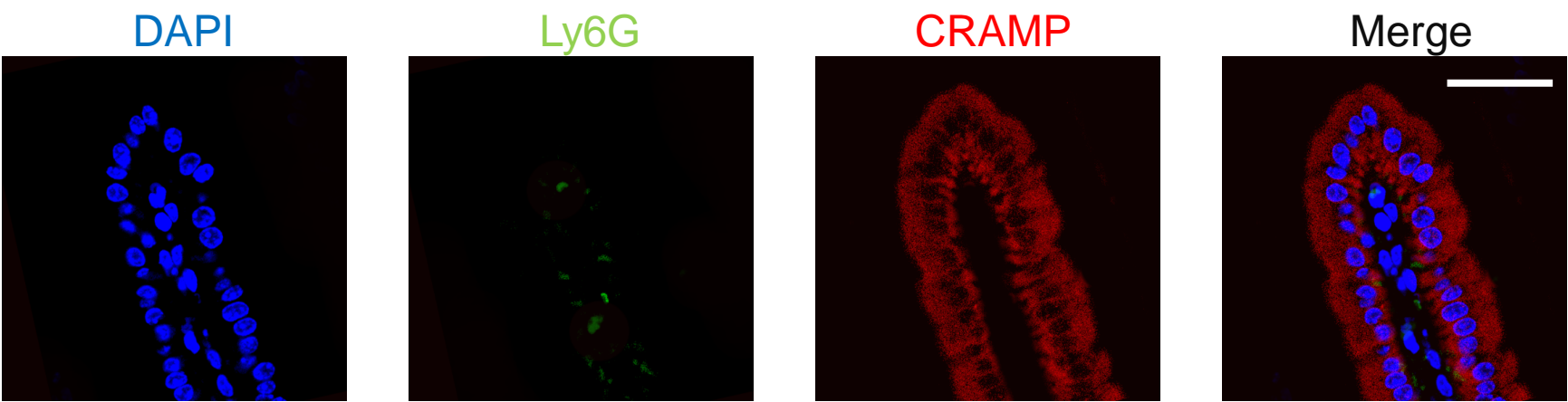

Figure 1E  
Gluten

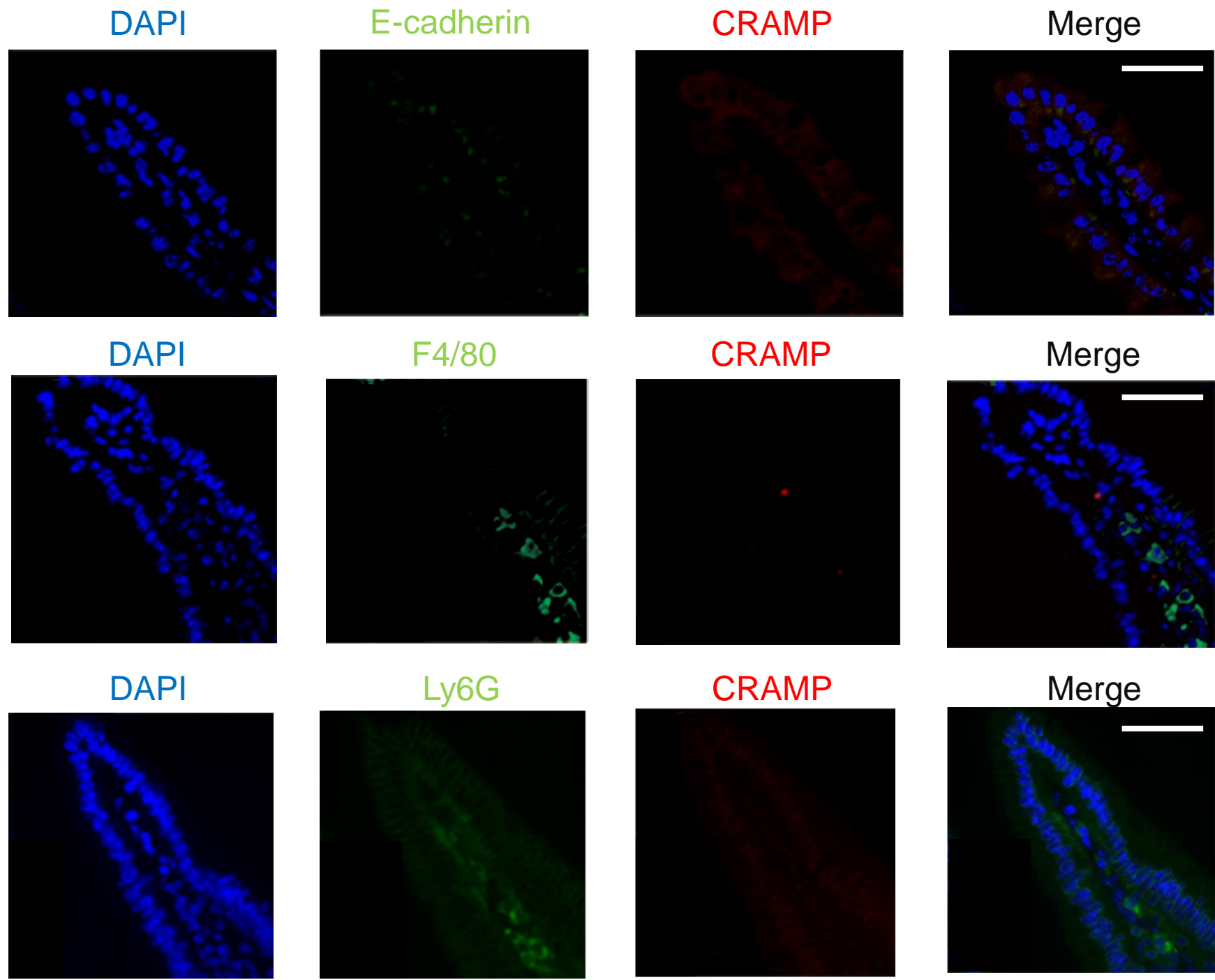

Supplement: Supplementary file 5 — Source Data for Figure 1 [file EMMM-13-e14059-s010.zip › EMM-2021-14059_SourceDataForFigure1C-E.pdf]

Figure 2D

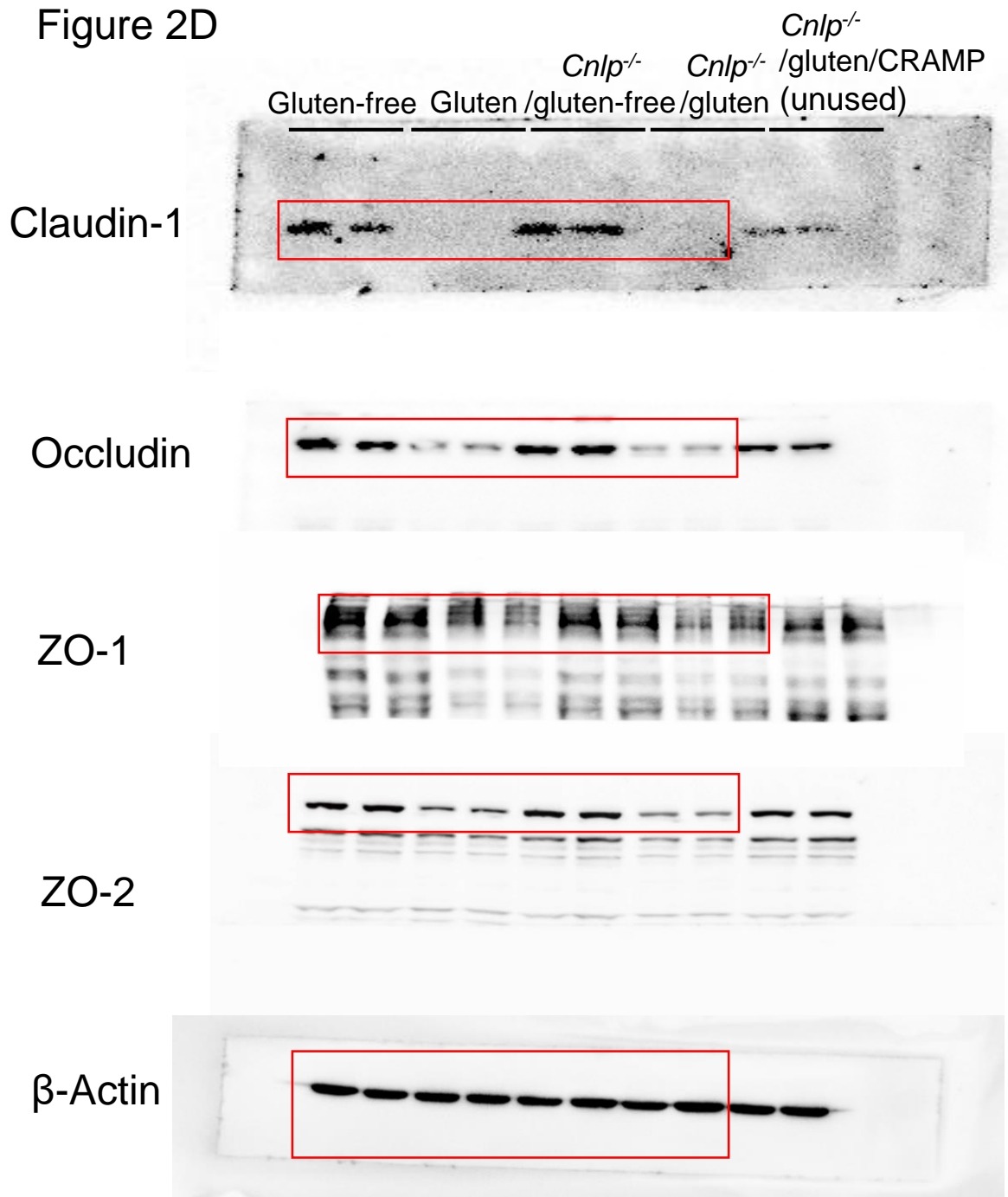

Figure 2E

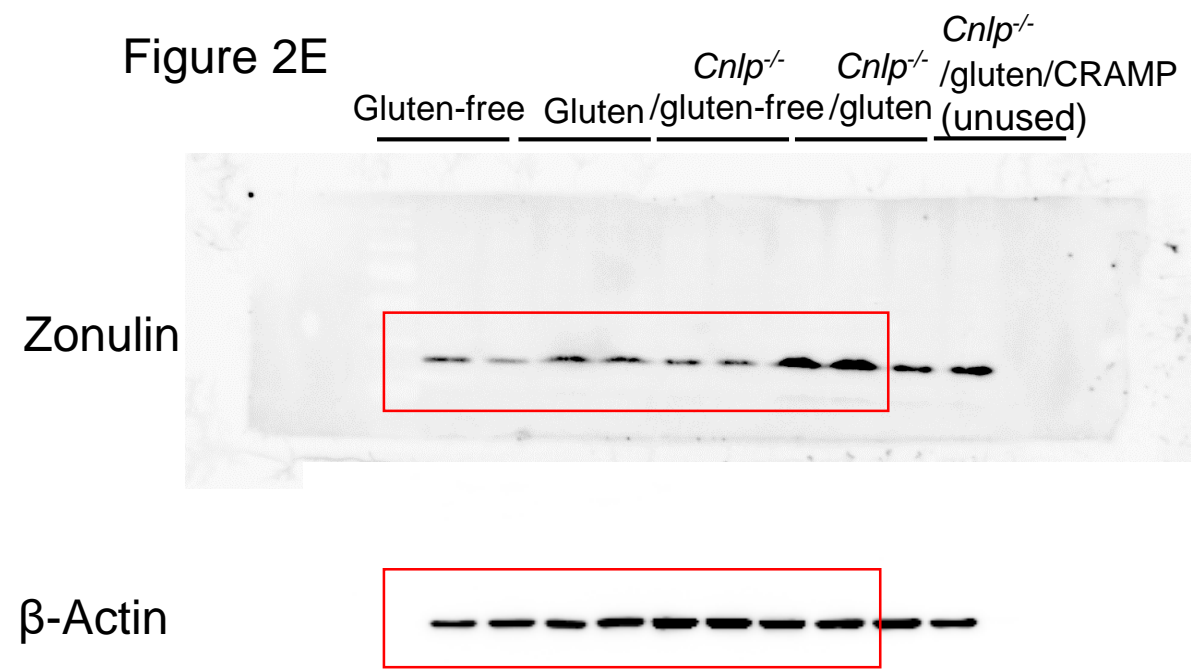

Supplement: Supplementary file 6 — Source Data for Figure 2 [file EMMM-13-e14059-s007.zip › EMM-2021-14059_SourceDataForFigure2D-E.pdf]

Figure 4A

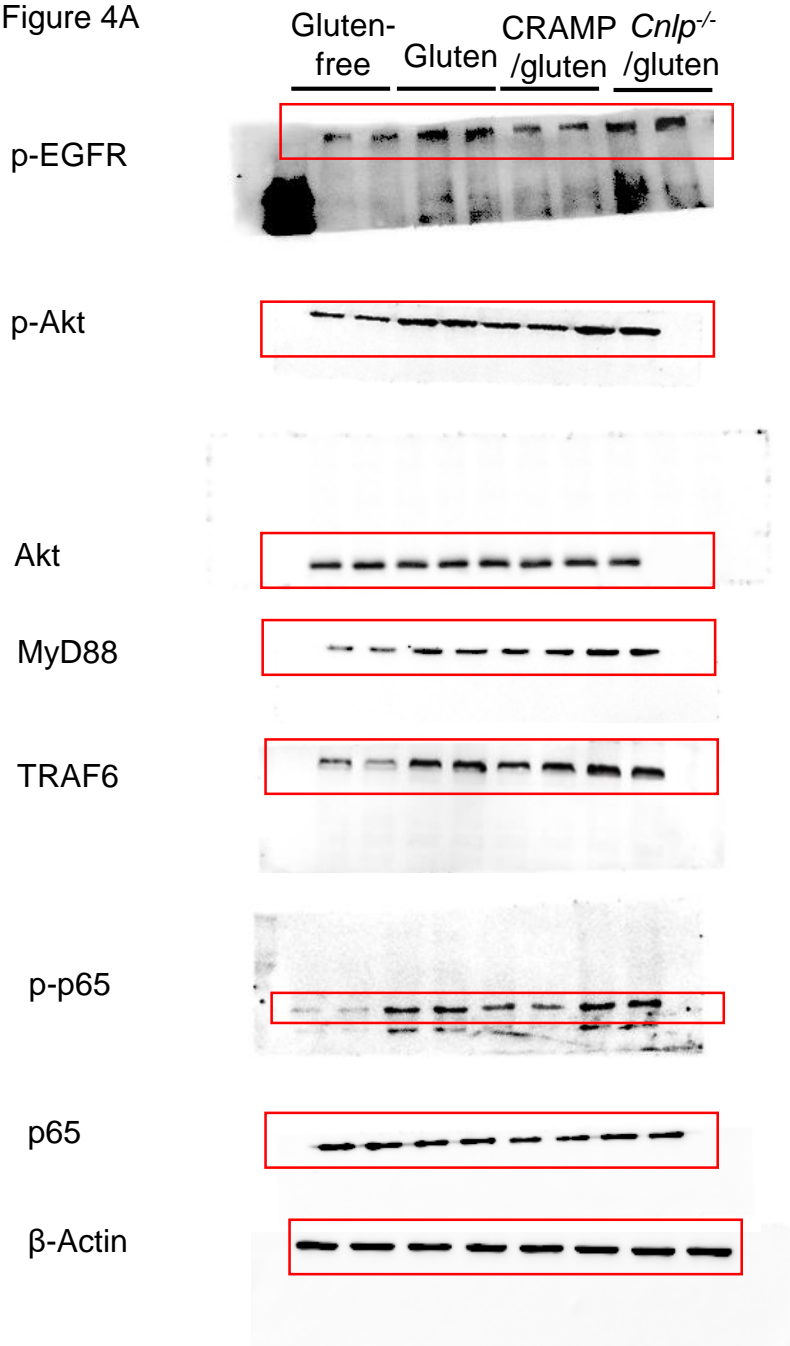

Figure 4B

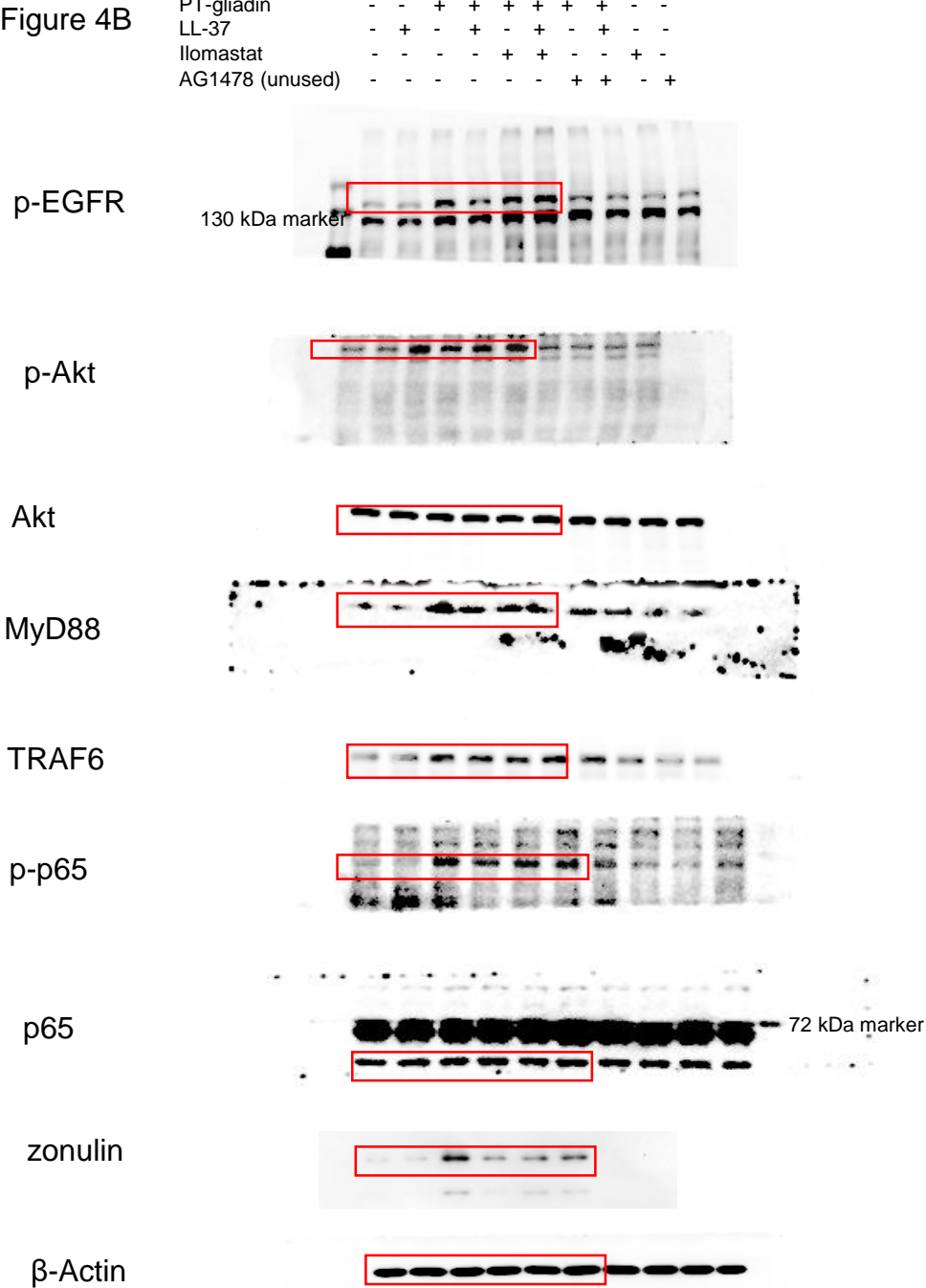

Supplement: Supplementary file 8 — Source Data for Figure 4 [file EMMM-13-e14059-s011.zip › EMM-2021-14059_SourceDataForFigure4A-B.pdf]

Figure 6I

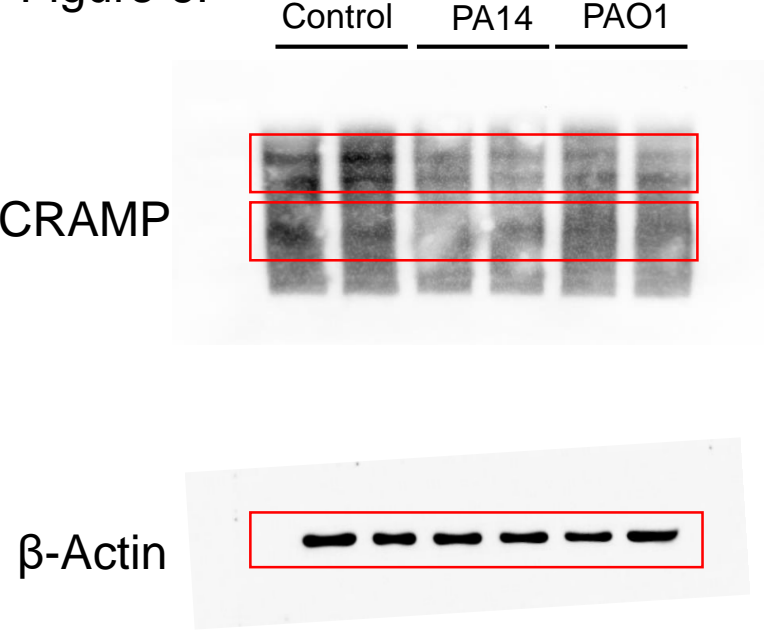

Supplement: Supplementary file 10 — Source Data for Figure 6 [file EMMM-13-e14059-s003.zip › EMM-2021-14059_SourceDataForFigure6I.pdf]

Figure 7C

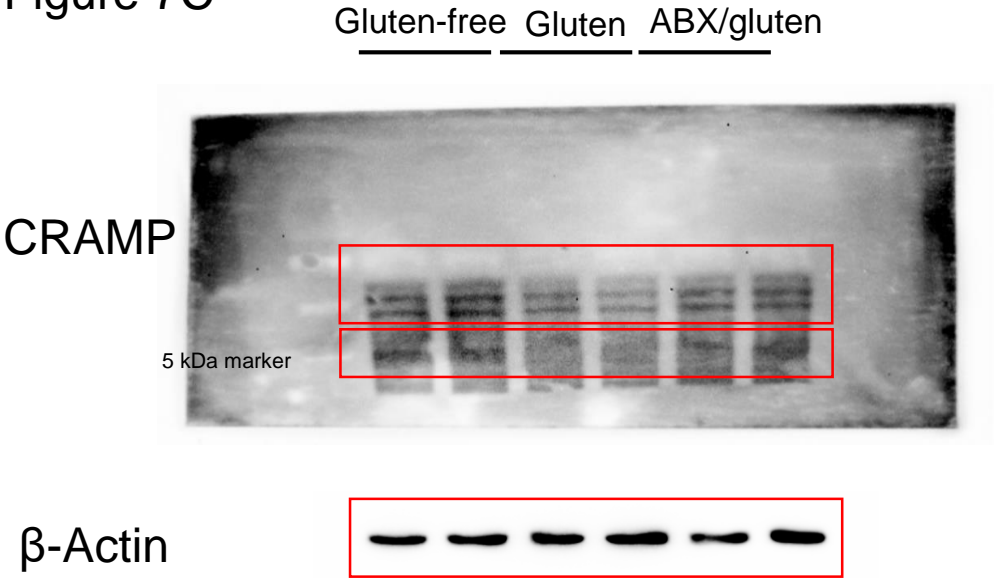

Figure 7E

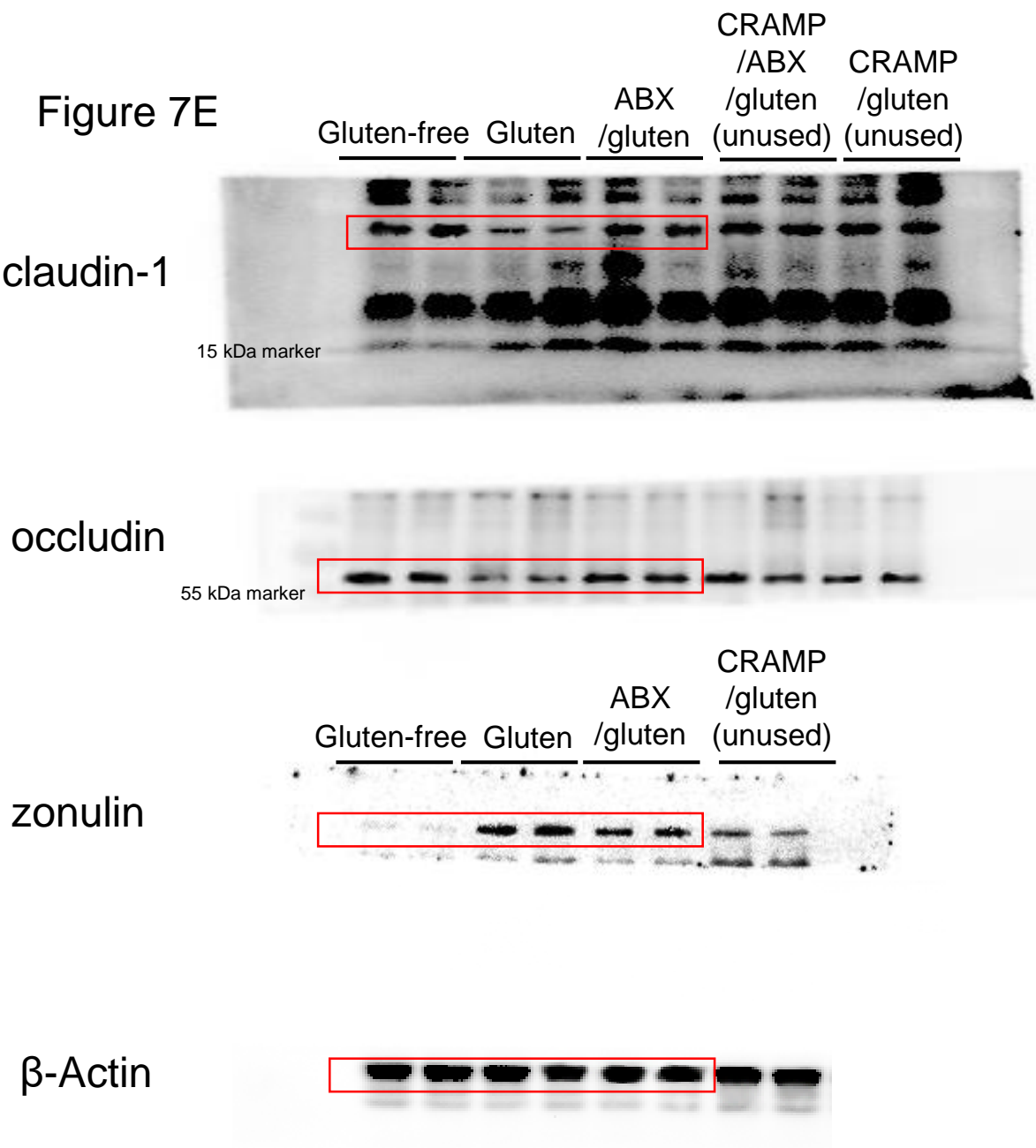

Supplement: Supplementary file 11 — Source Data for Figure 7 [file EMMM-13-e14059-s008.zip › EMM-2021-14059_SourceDataForFigure7CE.pdf]
