# Supplementary material for: Gut microbiota‐CRAMP axis shapes intestinal barrier function and immune responses in dietary gluten‐induced enteropathy
Source: EMBO Mol Med. 2021 Jun 14;13(8):e14059. doi: 10.15252/emmm.202114059 (PMC8350901; doi:10.15252/emmm.202114059)

Figure 3E

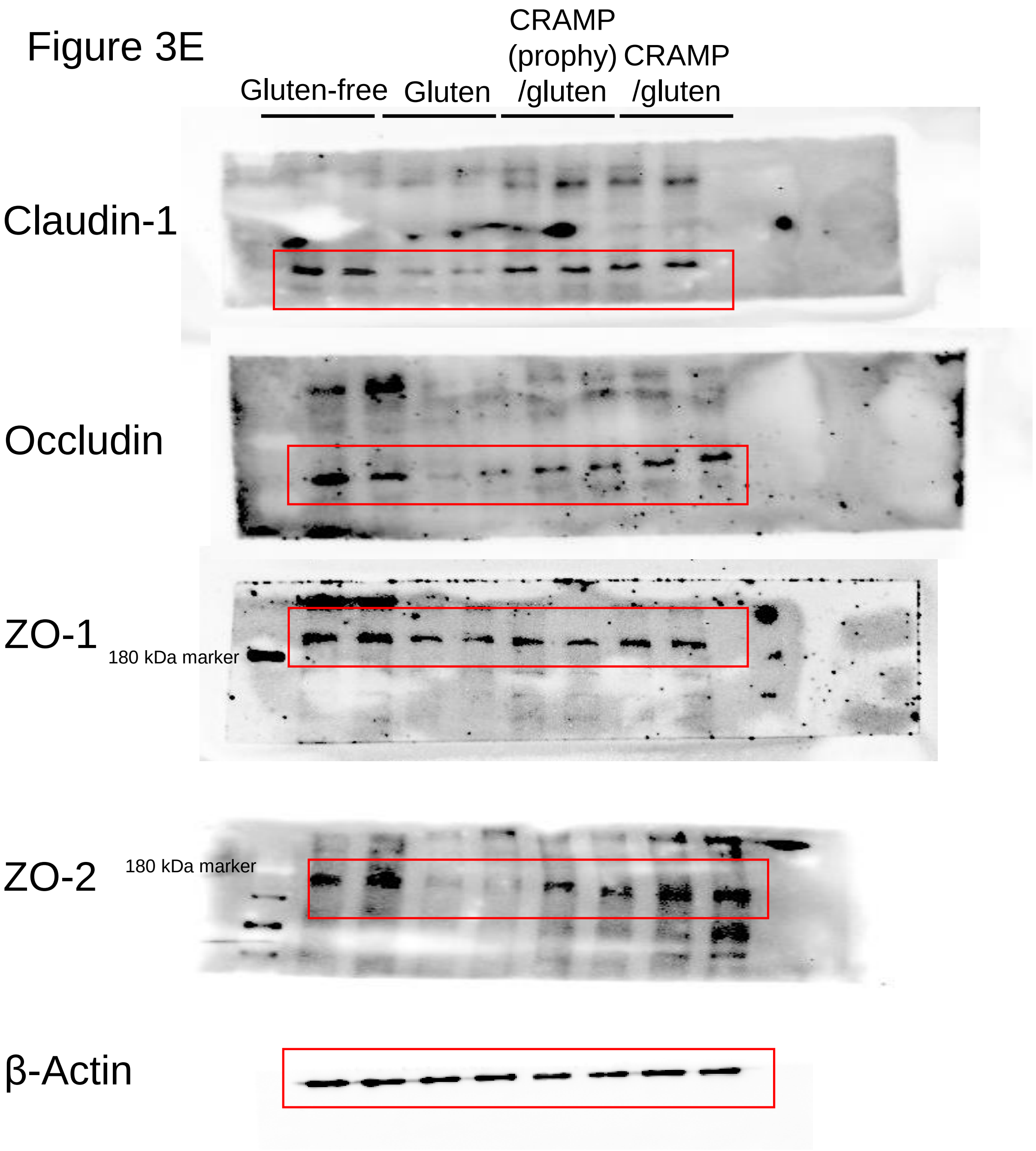

Figure 3F

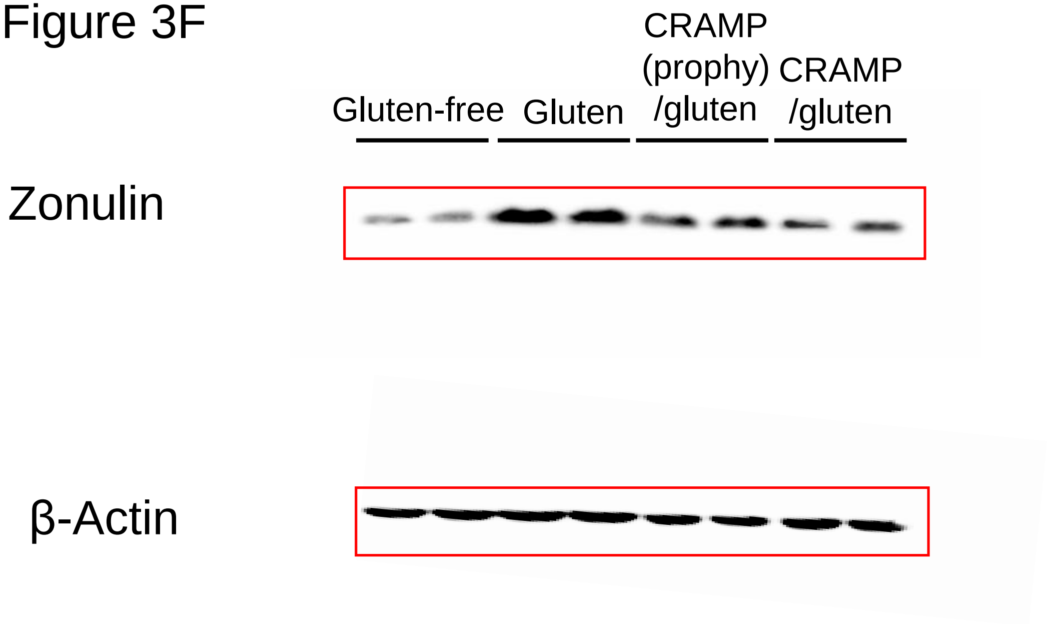

Supplement: Supplementary file 7 — Source Data for Figure 3 [file EMMM-13-e14059-s002.zip › EMM-2021-14059_SourceDataForFigure3E-F.pdf]
