# Supplementary material for: Gut microbiota‐CRAMP axis shapes intestinal barrier function and immune responses in dietary gluten‐induced enteropathy
Source: EMBO Mol Med. 2021 Jun 14;13(8):e14059. doi: 10.15252/emmm.202114059 (PMC8350901; doi:10.15252/emmm.202114059)
Supplement: Supplementary file 3 — Source Data for Expanded View [file EMMM-13-e14059-s001.zip › EMM-2021-14059_SourceDataForEVFig2.zip!/EMM-2021-14059_SourceDataForEVFig2.pdf]

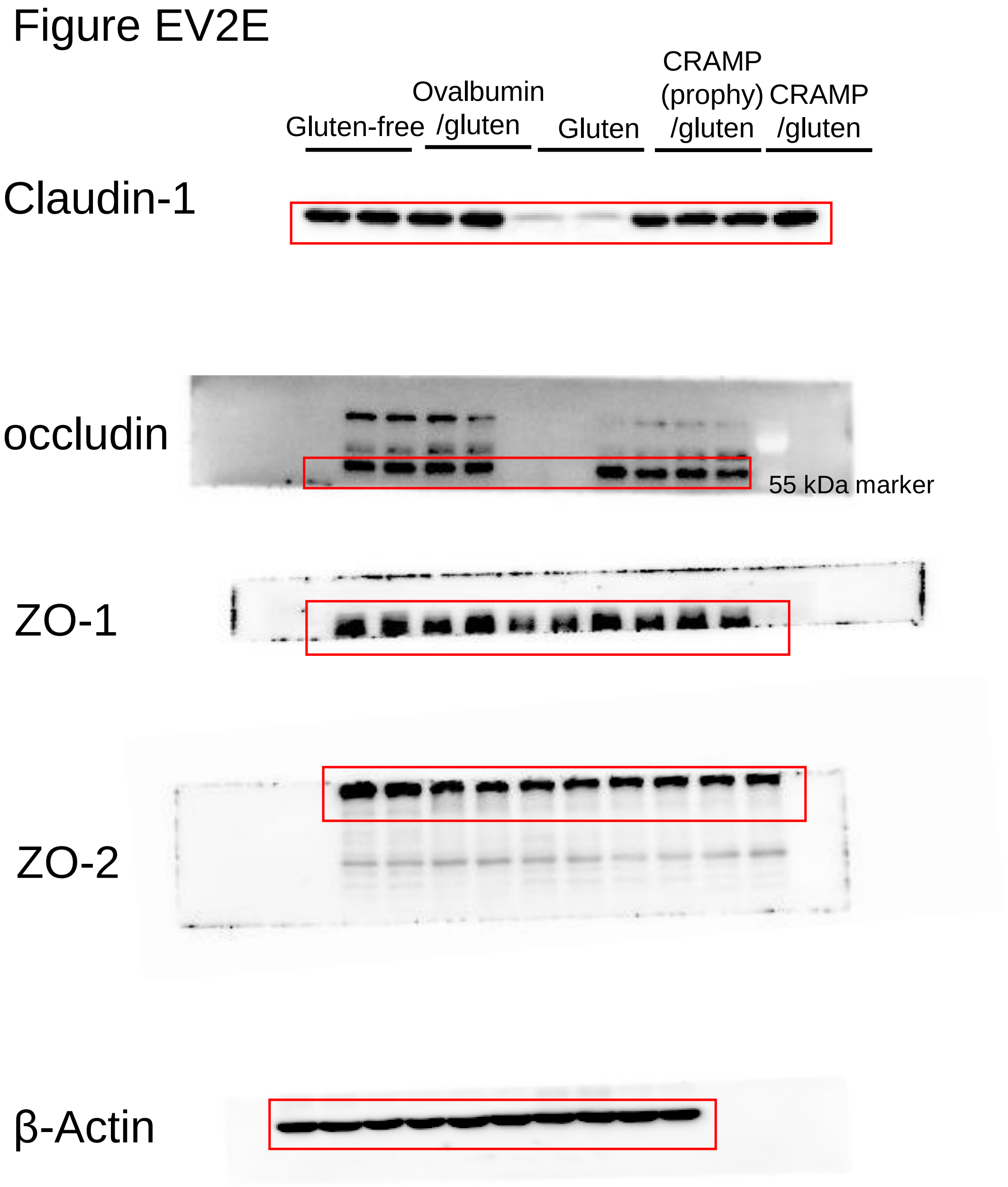
Figure EV2E
Gluten-free
Ovalbumin /gluten
Gluten
CRAMP (prophy) /gluten
CRAMP /gluten
Claudin-1
occludin
55 kDa marker
ZO-1
ZO-2
β-Actin

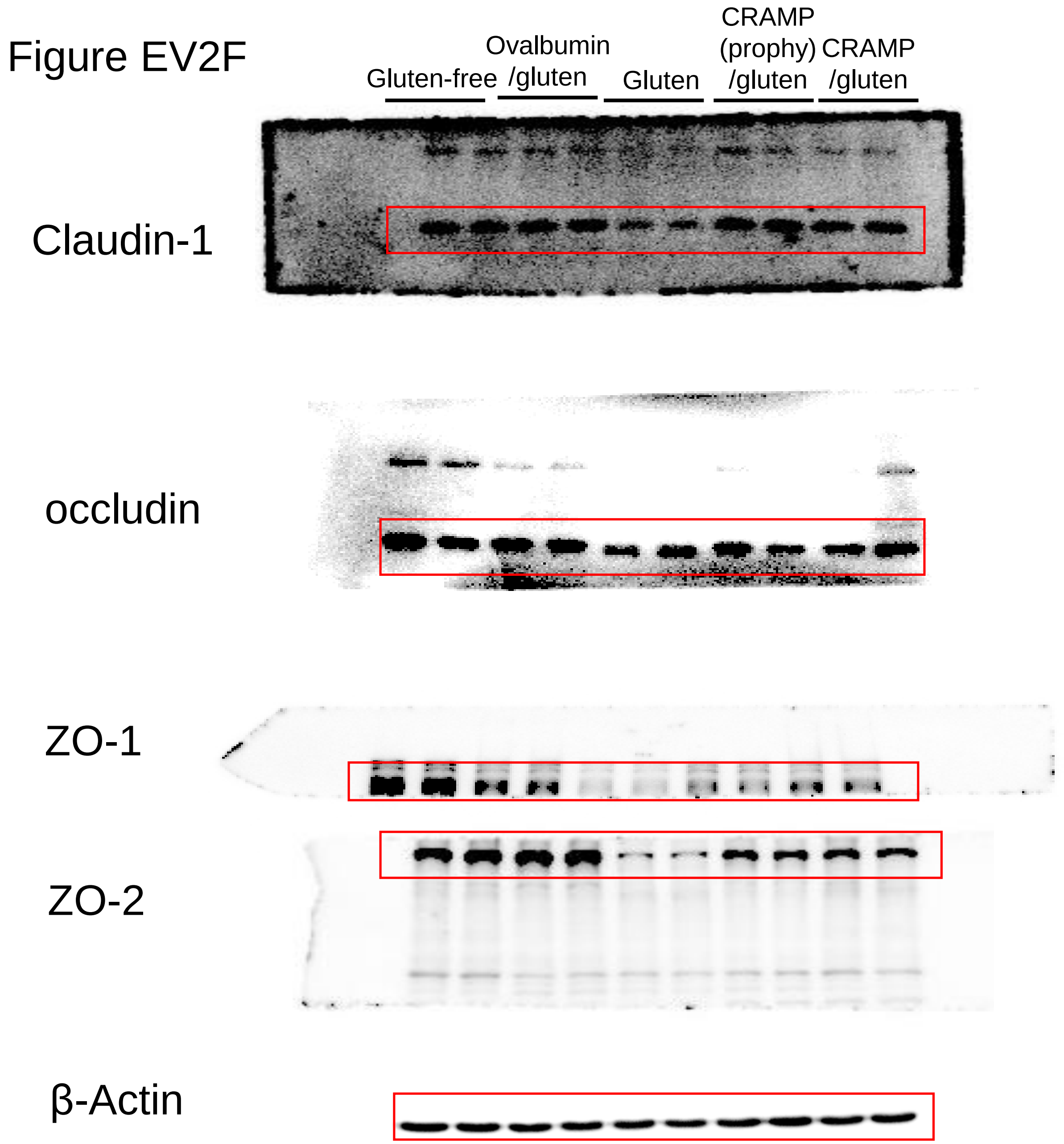
Figure EV2F
Gluten-free
Ovalbumin /gluten
Gluten
CRAMP (prophy) /gluten
CRAMP /gluten
Claudin-1
occludin
ZO-1
ZO-2
β-Actin
